# Intention to use maternal health services and associated factors among women who gave birth at home in rural Sehala Seyemit district: a community-based cross-sectional study

**DOI:** 10.1186/s12884-022-04447-y

**Published:** 2022-03-16

**Authors:** Birhan Tsegaw Taye, Azmeraw Ambachew Kebede, Kindu Yinges Wondie

**Affiliations:** 1 School of Nursing and Midwifery, Asrat Woldeyes Health Science Campus, Debre Berhan University, PO. Box 445, Debre Berhan, Ethiopia; 2 Department of Clinical Midwifery, School of Midwifery, College of Medicine and Health Sciences, University of Gondar, Gondar, Ethiopia

**Keywords:** Ethiopia, Home-delivery, Intention, Maternal health service utilization

## Abstract

**Background:**

Low maternal healthcare service utilization contributes to poor maternal and newborn health outcomes in rural Ethiopia. 'Motivational factors influence women's intention to perform a specific health behavior, and the intention of subsequent home delivery and related risks that may contribute to women's death is less known. Therefore, this study aimed to assess the intention of maternal health service utilization among women who gave birth at home in the rural Sehala Seyemit district.

**Methods:**

A community-based cross-sectional study was conducted from September 1^st^ to October 15^th^, 2020, among 653 women. A two-stage sampling technique was used to select the study participants. First, a semi-structured, pretested, and interviewer-administered questionnaire were used. The mean of the sum score was also used to categorize the intention as intended and not intended. Second, multivariable logistic regression analysis was computed to identify factors associated with women's intention to use maternal health services. Adjusted odds ratio (AOR) with a 95% confidence interval at a *p*-value of ≤ 0.05 were used to declare statistical association.

**Results:**

Of the women who gave birth at home the intention to use maternal health service was 62.3% (95% CI; 59, 66). Women’s age of > 30 years (AOR = 6.04; 95%CI: 2.34, 15.60), short time to reach health facility (AOR = 2.52; 95% CI: 1.57, 4.10), media exposure (AOR = 2.10; 95% CI: 1.16, 3.65), history of obstetric danger signs (AOR = 4.60; 95% CI: 2.33, 9.10), positive subjective norms (AOR = 11.20; 95% CI; 6.77, 18.50) and last delivery assisted by traditional birth attendants (AOR = 0.15; 95% CI: 0.06, 0.33) were factors associated with women’s intention to use maternal health services.

**Conclusion:**

In this study, maternal health service utilization intention is still unsatisfactory compared to the national target plan. Maternal age, media exposure, obstetric danger signs, distance to a health facility, positive subjective norms, and delivery assistant at delivery were predictors of women's intention to use maternal healthcare services. Improving women's awareness of maternal healthcare services and developing strategies to increase women's access to mass media, skilled birth attendants, and transportation for rural women may enhance their intention to use maternal healthcare services.

**Supplementary Information:**

The online version contains supplementary material available at 10.1186/s12884-022-04447-y.

## Introduction

Maternal Health Services (MHSs) refers to the health services provided to women during pregnancy, intrapartum, and postpartum. It caresses one of the health service strategic frameworks and indicators of maternal, newborn, children, and adolescents' health [1]. Indeed, utilization of these basic services is essential to reduce and manage pregnancy-related complications; in turn, this may help to drop the burden of preventable maternal and neonatal deaths, stillbirths, and the community consequences at large [2]. Unfortunately, many women still have little or no access to essential and good-quality maternal health services worldwide [1]. Ethiopia has also been suffering from a similar burden [3–5].

Reducing maternal mortality is a top public health priority as its magnitude is still high [6, 7]. About 830 women daily and 303,000 women annually died from pregnancy and childbirth-related complications globally by 2015 [8]. Nearly all of these deaths occurred in low-resource settings, including West and Central Africa (WCA), and Sub-Saharan Africa (SSA), and most of these deaths could have been preventable [8]. According to United Nations Children's Fund (UNICEF) 2017 report, the Maternal Mortality Ratio (MMR) was 216 worldwide, 679 in WCA, and 546 in SSA per 100 000 live births [9].

Evidence-based strategies and quick actions are compulsory to reduce such an unacceptably high maternal mortality ratio. In this context, strengthening and improving MHSs utilization is among the key strategies of achieving the Sustainable Development Goal (SDG) 2030 target (i.e., less than 70 maternal deaths per 100,000 live births) and ultimately eliminating preventable maternal mortality [7]. Ethiopian Health Sector Transformation Plan II report showed that MMR was 401/100,000 live births in 2017. Besides, under-5 mortality and infant mortality per 1000 live births reduced from 123 and 77 in 2005 to 59 and 47, respectively, in 2019. However, there have been no significant reductions in neonatal mortality (33 deaths per 1,000 live births in 2019) [10]. In connection to this, 2019 mini EDHS data shows that only 43% of the women received four or more antenatal care (ANC) visits from a skilled provider, 52% of the births took place at home without the assistance of trained persons, and 34% of women received PNC check-up in the first two days after birth [11]. Thus, Ethiopia has adapted the current global agenda and developed many strategies by encouraging the utilization of MHS to reinforce maternal health components.

Maternal healthcare services (MHSs) utilization is a complex behavioral phenomenon in nature and mainly determined by an individual's behavioral intention, which in turn, is determined by personal evaluations of pros and cons (i.e., attitude), the beliefs and behaviors of important others (perceived social norm) and personal capabilities (i.e., perceived behavioral control/self-efficacy) [12, 13]. Understanding women's intention of health-seeking behavior is a supreme power in the design of public health interventions for improving poor maternal and newborn health outcomes [14].

Despite the national struggles to increase maternal healthcare uptake, the percentage of home deliveries remains higher in Ethiopia, particularly in the rural community [15, 16]. Previous studies examined women's intention to MHSs utilization. However, none of these studies reached out to and reviewed the intention of women who underwent home delivery to assess all aspects of their future maternal healthcare-seeking behavior.

Given the limited utilization of MHSs in Ethiopia, it is important to examine the women's behavioral intention and influencing factors to use MHSs as the intention has the most immediate influence on behavior to perform a certain task. Therefore, this study aimed to assess the health-seeking intentions of women and to identify individual-level factors in the Sehala Seyemit district. Besides, doing the research may offer basic evidence for any interventions aimed at improving MHSs utilization within the community context and to design effective strategies towards tackling preventable maternal and newborn morbidity and mortality.

## Methods and materials 

### Study design, setting, and period

A Community-based cross-sectional study was conducted from September 1^st^ to October 15^th^, 2020. This study was conducted in rural Sehala Seyemit district, Waghimra zone, Amhara regional state, northern Ethiopia. Sehala Seyemit district is located 285 km northeast of Bahir Dar (the capital city of Amhara regional state) and about 799 km north of Addis Ababa (the capital city of Ethiopia). Accessing health services in the district is difficult because of the lack of transportation to each "*kebeles*" (which is the smallest administrative unit in Ethiopia). The district has 13 "*kebeles*"; 12 rural and one urban "kebeles". Currently, the district has a population of about 39,435. Almost 90% of the population are farmers. There are three health centers and 13 health posts serving the community.

### Study population

During the data collection period, the study population was all women who gave birth at home in the last two years in the selected "kebeles". Critically ill women throughout the data collection period were excluded.

### Sample size determination and sampling procedure

The sample size for this study was determined by using a single population proportion formula by considering the following assumptions: women's intention to deliver in a health facility 30.3% [17], 95% level of confidence, and 5% margin of error.

$$\mathrm{n}=\frac{Z\alpha /2{)}^{2}p(1-p)}{{d}^{2}}=\mathrm{n}=\frac{(1.96{)}^{2}* 0.303*(1-0.303)}{(0.05)2}=325$$

By considering a design effect of 2 and a 5% non-response rate; (i.e., adding 5% of 325 = 325 + 16.25 and multiplying by two), the minimum adequate sample size was 683.

Stud participants were selected using a multi-stage sampling technique. Eight kebeles were selected randomly in the first stage among the 12 "*kebeles*". The list of the study participants was gained from health extension workers (HEWs) and local administrators. Next, we designed a sampling frame by numbering the list of women. Then the total sample size was distributed in proportion to the size of each selected "*kebeles*". Lastly, the study participants were selected by a simple random sampling technique using a random generation table.

### Variables of the study

#### Outcome variable

Intention to use maternal health services (Intended/ not intended).

#### Independent variables

Age of the women, marital status, women's educational status, women's occupation, husband educational status, husband occupation, family size, exposure to mass media, time to reach the nearby health facility, parity, history of ANC, number of ANC, birth assistant, history of PNC, husband involvement in maternal and children's health, household decision-making power, history of abortion, history of neonatal death, history of obstetric danger signs during the recent pregnancy, status of the pregnancy, knowledge of MHS, subjective norms, perceived behavioral control, and attitude towards MHS utilization.

### Measurements

#### Home delivery

Is when women deliver at home or birth in a residence rather than a birthing center without a skilled birth attendant [18].

#### Intention to use maternal health services

Women's intention to use MHS was measured using five questions: 1) Intention to use ANC; 2) Intention to use MWHs; 3) Intention to pay for ANC/ delivery if it is with a cost; 4) Intention to deliver in a health facility 5) Intention to use postnatal care (PNC). Each question has five-point Likert scales (1 = strongly disagree, 2 = disagree, 3 = neutral, 4 = agree, 5 = strongly agree). The total score was ranged from 5–25 and women who scored approaching the maximum score of the total item were considered to have good intentions to use maternal health services. The mean of the sum score was also used to categorize the intention as intended and not intended if women were scored above mean and below mean respectively [19].

#### Women’s attitude

This refers to the extent to which an individual has a positive or negative estimation of the behavior of interest. Women's attitude towards MHS utilization was measured using 13 questions: 1) All pregnant women should have ANC and PNC follow up; 2) Pregnant and lactating women should use a variety of foods rich in protein and vitamins; 3) The healthcare provided by health providers is important; 4) Timely ANC follow-up will be safer for both mother and baby during labor and delivery; 5) Taking medication during pregnancy without a doctor's prescription can cause problems for the fetus; 6) Husbands should be present during ANC, delivery and PNC; 7) It is important to be prepared during pregnancy; 8) Maternity waiting homes are very important for women far from health facilities; 9) Heavy weight lifting and strenuous exercise during pregnancy is dangerous and may be unsafe for the fetus; 10) Advice regarding proper health during pregnancy and childbirth can be found outside the hospital; 11) Follow up during pregnancy may decrease intrapartum and postpartum complications; 12) Health facility delivery is safer and better than home delivery; 13) Women may have problems without ANC, health facility delivery and PNC. Each question has five points Likert scale (1 = strongly disagree, 2 = disagree, 3 = neutral, 4 = agree, 5 = strongly agree). The total score was 13–65, and women who scored above the mean were considered to have a favorable attitude [20, 21].

#### Subjective norms towards maternal health utilization

This refers to whether the majority of people support or reject the behavior. It was measured using five questions: 1) People who are important to me think that I should use antenatal care during pregnancy; 2) Important people to me think that I should use maternal waiting homes in the last 2–4 weeks of my pregnancy; 3) People who are important to me think that I have to deliver in a health facility; 4) Important people to me think that I have to get a skilled birth attendant during delivery; 5) Important people to me think that I should follow postnatal care and immunization services. Each question has five-point Likert scales (1 = strongly disagree, 2 = disagree, 3 = neutral, 4 = agree, 5 = strongly agree). The minimum and maximum scores were 5 and 25, respectively. Women who scored above the mean value were considered positive subjective norms.

#### Perceived behavioral control

This refers to an individual's perception of the simplicity or complexity of performing the behavior of interest. It was measured using five questions: 1) For me attending antenatal care is simple, and I can do it; 2) For me using maternity waiting homes in the last 2–4 weeks of my pregnancy is simple, and I can do it; 3) For me health facility delivery is very simple, and I can do it; 4) Getting a skilled birth attendant is easy for me and I can do it; 5) Using postnatal and immunization services are easy to me, and I can do it. The cumulative score ranged from 5 to 25 and women who scored above the mean value were considered to have positive subjective norms.

#### Knowledge of maternal health services

Includes knowledge of ANC, PNC, and pregnancy, knowledge of obstetric danger signs, knowledge of birth preparedness and complication readiness, knowledge of malaria prevention, knowledge of anemia prevention, knowledge of helminthic infection prevention, knowledge of tetanus prevention during pregnancy and knowledge of complications of home delivery. The study participants were asked 16 questions (See Additional file 1). These items comprise "Yes" or "No" and multiple response options. One point was given for all the correct answers, while zero point for incorrect answers. Then, women's knowledge was composed (knowledgeable which was coded as "1" and not knowing which was coded as "0"). Thus, based on the summative score of variables designed to assess knowledge with a score above the mean was considered as knowledgeable and vice versa [20–22].

#### Husband involvement

Husband involvement in maternal and child health-related activities was measured using nine questions: 1) Did your husband go with you for ANC follow-up at least once in your most recent pregnancy? 2) Did your husband provide transport/give money for transport during your recent pregnancy or delivery? 3) Did your husband accompany you to the hospital during labor for your recent delivery? 4) Did your husband discuss with health care providers during your recent pregnancy or delivery? 5) Did your husband look after the child at home/stay with the babies while you are outside the home? 6) Did your husband bathe a newborn/infant while you were busy? 7) Did your husband buy clothes/other things for infants/neonates? 8) Did your husband go with you for immunization services? 9) Did your husband assist you while you breastfed the newborn/infant? Each question was coded as 0 for "no" and 1 for "yes". The total score ranged from 0–9, and a score above the mean was considered as husband involved [23, 24].

#### Household decision-making power

Household decision-making power was assessed using nine questions: 1) who decides about health care for you? 2) Who decides on the large household purchase or sell? 3) Who decides on intra-household resource allocation/ daily household purchases? 4) Who decides where and when to seek medical care for sick newborns/children? 5) Who decides on visits of family, friends, or relatives? 6) Who decides when to have an additional child? 7) Who usually decides how your partner's/husband earnings will be used? 8) Who decides to go for ANC, PNC, where to deliver, and infant immunization? 9) Who usually decides what foods to be cooked each day? The possible answers were me alone, which was coded as 2, both of us which was coded as 1, the husband alone, which was coded as 0. The score ranged from 0 to 18 and a woman who scored above the mean was considered to have higher household decision-making power [25].

### Data collection instruments, procedures, and quality control

The data collection tool was developed by reviewing the literature [17, 26, 27] and collected using a semi-structured, interviewer-administered questionnaire through face-to-face interviews ( See Additional file 2). First, the questionnaire was prepared in English, translated into Amharic, and returned to English to confirm consistency. The questionnaire contains questions about socio-demographic characteristics, reproductive and childbirth-related health care characteristics, women's knowledge of MHS, attitudes towards maternal health services, and their intention to use MHS. Before the actual data collection, we did a pretest on 5% of the calculated sample size (34 women) at Ziquala Woreda, which has similar socio-cultural and living standards as the study area. The necessary amendments were done accordingly. Eight female HEWs and four male midwifery diploma holders were recruited for data collection and supervision. Two-day training was given regarding the overall data collection process. At the time of data collection, the questionnaire was checked for completeness daily by the supervisors. Then, feedback was given to data collectors.

### Data processing and analysis

The data collected from the field were checked for completeness and consistency, coded and entered into Epi-info version 7.1.2, and exported to SPSS version 25 computer software package for analysis. Frequencies and cross-tabulations were used to summarize descriptive statistics. Texts, tables, and graphs presented the data. Moreover, binary logistic regression was used to identify factors associated with the future intention of home-delivered women on MHS utilization. Then variables were fitted to multivariable logistic regression using the backward likelihood ratio method. Both COR and AOR with 95% CI were computed to show the strength of the association. Finally, a statistically significant association of variables was declared based on AOR with 95% CI and *p*-value < 0.05.

## Result

### Socio-demographic characteristics of study participant

This study included six hundred fifty-three women, giving a 97.5% response rate. The mean age of the respondents was 26.4 years (SD ± 4.93), and more than two-thirds of the participants were aged between 21 and 30 years. Most (97.2%) of the participants were married, and 384/653 (58.8%) of them could not read and write. Over four-fifth (81.6%) of the women were farmers by occupation. Regarding husband occupation, 85% were farmers, and slightly more than half (51%) of them could not read and write (Table1).

**Table 1:** Socio-demographic characteristics of study participants in Sehala Seyemit district, northern Ethiopia, 2020 (*n* = 653)

Characteristics	Category	Frequency	Percentage (%)
Age of women in year	≤ 20	70	10.7
	21–30	440	67.4
	≥ 31	143	21.9
Current marital status	Married	635	97.2
	Unmarried	18	2.8
Family size	2–3	84	12.9
	4–6	433	66.3
	≥ 7	136	20.8
Women’s educational status	Unable to read and write	384	58.8
	Able to read and write	254	38.9
	Primary education	15	2.3
Women’s occupation	Famer	533	81.6
	Merchant	89	13.6
	Others ^a^	31	4.8
Husband educational status (*n* = 635)	Unable to read and write	235	37
	Able to read and write	324	51
	Primary education and above	76	12
Husband occupation (*n* = 635)	Famer	540	85
	Merchant	92	14.5
	Others ^a^	3	0.5
Exposure to mass media	Yes	433	66.3
	No	220	33.7
Type of transport at the time of emergency	On foot/traditional ambulance	426	65.3
	Ambulance	142	21.7
	Private bus	85	13

*Note*: ^a^ = student and daily laborrer

### Reproductive history, maternity healthcare service, and behavioral related characteristics

Only 198/653 (30.3%) of the women had at least one ANC visit in their recent pregnancy. More than half (56.5%) of the participants had adequate knowledge of MHS. About three-fourths (75.2%) of the women had a positive attitude towards service utilization (Table 2).

**Table 2:** Reproductive, maternity healthcare service and behavioral characteristics of study participants in Sehala Seyemit district, northern Ethiopia, 2020 (*n* = 653)

Characteristics	Category	Frequency	Percentage (%)
Parity	1	74	11.3
	2–4	436	66.8
	≥ 5	143	21.9
Had ANC	Yes	198	30.2
	No	455	69.8
Number of ANC follow-up (*n* = 198)	< 4	191	96.5
	≥ 4	7	3.5
Birth assistant for the recent birth	HEWs	379	58
	TBA	204	31.3
	Family members	70	10.7
Had postnatal care	Yes	82	12.6
	No	571	87.4
Husband involvement in maternal and child health	Involved	363	56.6
	Not involved	290	44.4
History of abortion	Yes	63	9.6
	No	590	90.4
History of neonatal death	Yes	19	2.9
	No	634	97.1
Experienced obstetric danger signs in the most recent pregnancy	Yes	119	18.2
	No	534	81.8
Time to reach a health facility	< 1 h	326	49.9
	≥ 1 h	327	50.1
Household decision-making power	Higher	495	75.8
	Lower	158	24.2
Status of pregnancy	Planned	573	87.7
	Unplanned	80	12.3
Knowledge of maternal health services	Knowledgeable	369	56.5
	Not knowledgeable	284	43.5
Attitude towards maternal health services	Favorable	491	75.2
	Unfavorable	162	24.8
Subjective norms	Positive	285	43.6
	Negative	368	56.4
Perceived behavioral control	Positive	204	31.2
	Negative	449	68.8

### Women's intention to use maternal health services

**Fig. 1:**
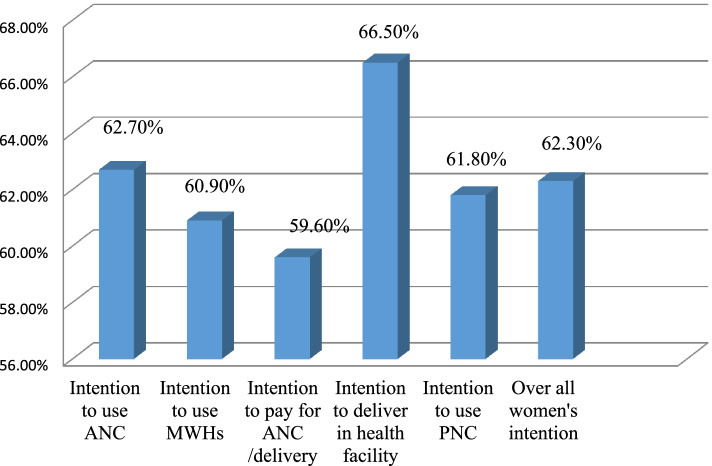
Intention to use maternal health services among women who gave birth at home in rural Sehala Seyemit diistrict Northern Ethiopia, 2020

Overall, nearly two-thirds (62.3%) of women were willing to use MHS for their future pregnancies (95% CI; 59, 66). About 62.7% of women intended to use antenatal care for their upcoming pregnancies. Slightly more than two-thirds (66.5%) of the participants intended to give birth at a health facility (Fig. 1).

### Factors associated with women's knowledge of antenatal care

Multivariable logistic regression analysis revealed that older maternal age, most recent birth assisted by traditional birth attendants (TBA), short time to reach the nearby health facility, experiencing obstetric danger signs in the most recent delivery, positive subjective norms, and mass media exposure were factors associated with intention to use MHSs.

Accordingly, this study found that those women older than 30 and 21–30-year-old were six (AOR = 6.04; 95% CI: 2.34, 15.60) and 2.57 (AOR = 2.57; 95% CI: 1.25, 5.28) times more likely to have an intention to utilize MHSs for the upcoming pregnancy as compared to women below 20 years of age, respectively.

This study also established that women who traveled for less than one hour were 2.52 times more likely to have an intention to use MHSs as compared with those women who traveled for more than an hour (AOR = 2.52; 95% CI: 1.57, 4.10).

The odds of having an intention to MHSs utilization were two times higher among women who had been exposed to mass media as compared with the reference group (AOR = 2.10; 95% CI: 1.16, 3.65).

This study also shows that participants who experienced obstetric danger signs in their recent pregnancy were four times more likely to intend to use MHSs as compared to their counterparts (AOR = 4.60; 95% CI: 2.33, 9.10).

The odds of having an intention to utilize MHSs were 11.2 times higher among women who had positive subjective norms than those who had negative subjective norms (AOR = 11.20; 95% CI: 6.77, 18.50). Likewise, women whose recent birth was assisted by traditional birth attendants were 85% less likely to have an intention to use MHSs as compared with their counterparts (AOR = 0.15; 95% CI: 0.06, 0.33).

Moreover, variables like women's attitude, parity, family size, positive perceived behavioral control, and higher decision-making power show an association in the bivariable logistic regression model. For example, the odds of intention to use MHS among women who had positive perceived behavioral control and higher decision-making power were 1.75 and 1.49 times higher than their counterparts, respectively. However, a significant association was not seen in the multivariable logistic regression analysis [Table 3].

**Table 3:** Bi-variable and multivariable logistic regression analysis of factors associated with intention to use MHS among women delivered at home in rural Sehala Seyemit district, northern Ethiopia, 2020 (*n* = 653)

Variables	Category	Intention to use maternal health services		COR (95%CI)	AOR (95%CI)
		**Yes**	**No**		
Women’s attitude	Favorable	329	162	2.2 (1.52, 3.14)	0.49 (0.25, 1.98)
	Unfavorable	78	84	1	1
Women’s perceived behavioral control	Positive	145	59	1.75 (1.23, 2.50)	0.99 (0.6, 1.6)
	Negative	262	187	1	1
Subjective norms	Positive	252	33	10.5 (6.9, 15.9)	11.2(6.77, 18.5) ^**^
	Negative	155	213	1	1
Age in year	≤ 20	31	39	1	1
	21–30	257	183	1.77 (1.1, 2.94)	2.57 (1.25, 5.28) ^*^
	> 31	119	24	6.24 (3.27, 11.9)	6.04 (2.34, 15.6) ^*^
Parity	1	42	32	1	1
	2–4	251	185	1.03 (0.63, 1.7)	2.0 (0.94, 4.25)
	≥ 5	114	29	2.91 (1.62, 2.54)	1.2 (0.47, 2.93)
Family size	2–3	47	37	1	1
	4–6	251	182	(0.68, 1.69)	0.9 (0.49, 1.69)
	≥ 7	109	27	(1.74, 5.81)	0.62 (0.27, 1.41)
Decision-making power	Higher	320	175	1.49 (1.04, 2.15)	0.36 (0.20, 1.7)
	Lower	87	71	1	1
Delivery assistant	HEWs	277	102	0.87 (0.48, 1.57)	0.67 (0.33, 1.4)
	TBA	77	127	0.19 (0.11, 0.36)	0.15 (0.06, 0.33) ^*^
	Family members	53	17	1	1
Time to reach health facility	< 1 h	238	89	2.48 (1.79, 3.44)	2.52 (1.57, 4.1) ^**^
	≥ 1 h	169	157	1	1
History of obstetric danger signs	Yes	99	20	3.63 (2.18, 6.1)	4.6 (2.33, 9.10) ^**^
	No	308	226	1	1
Media exposure	Yes	308	125	3.01 (3.15, 4.22)	2.1 (1.16, 3.65) ^**^
	No	99	121	1	1

*Notes*: * *p*-value < 0.05, ** *p*-value ≤ 0.001

## Discussion

The current study assessed intention to use MHSs for their subsequent pregnancy and associated factors among women who gave birth at home in rural Sehala Seyemit district, northern Ethiopia. This is the first study reaching out to women who gave birth at home and describes their intention to use MHSs. Overall, nearly two-thirds (62.3%) of the respondents had an intention to use MHSs. Moreover, women's intention to use MHSs was influenced by maternal age, birth assistant for the recent delivery, time to reach the nearby health facility, obstetric danger signs in the most recent pregnancy, positive subjective norms, and mass media exposure.

Accordingly, intention to use MHSs, include ANC (62.7%), MWH (60.9%), institutional delivery (66.5%), and PNC (61.8%). The overall intention to use MHSs in the current study is higher than local reports in Ethiopia, such as intention to use MWH in the Gurage zone-at 55.1% [28], and an intention to undergo health facility delivery in the Afar region- at 30.3% [17]. Moreover, a study conducted in Kenya, in which 51.8% of women were intended to use PNC services [29]. This might be connected to the government's involvement in the community mobilization and financial support for providing Maternal and Child Health (MCH) services.

On the other hand, this study revealed that the intention to give birth in a health facility is lower lower than studies conducted elsewhere in Ethiopia, including 74.3% of women in Debre Markos [30], 75% of women in Wollaita Soddo [27] were intended to give birth in a health facility. This could be justified as differences in the study setting and study population. The studies in Debre Markos and Wollaita Soddo were conducted in the urban community, whereas the current study was conducted in rural areas where MHS information may not be accessible. In addition, the aforementioned studies were conducted among ANC attendants (i.e., participants were already enrolled in the service). However, the current study was conducted in a rural area among women who gave birth at home. Empirical evidence shows that rural residence is independently associated with poor health care access [31, 32].

The current study found that the time it takes to get to the nearest healthcare facility was significantly associated with using MHSs. For example, it has been shown that women who traveled less than an hour to reach a health facility were 2.52 times more likely to use MHSs than women who traveled for more than an hour. This finding is consistent with studies conducted in Ethiopia [33, 34], Eretria [35], Kenya [29], and Zambia [36]. This could be justified by the fact that a long travel time or distance to a health facility is one of the well-known barriers to health care access especially, in developing countries [37]. In this regard, strengthening the HEWs program and investing in transport infrastructure should be a priority area for authorities and other stakeholders in charge. In Ethiopia's healthcare system, HEWs are the most nearby healthcare providers to the rural communities. In this way, supportive training and education can increase HEW's knowledge and motivation to serve the marginalized rural community, thereby enhancing women's willingness to use MHS through continuous health education.

The present study affirmed that utilization of TBAs for the recent birth had a significant association with women's intention to use MHSs. Thus, women whose preceding birth was assisted by TBAs were 85% less likely than women assisted by family members to be willing to use MHSs. Available evidence supplemented that women assisted by TBAs are less likely to use MHSs [38]. Even in the presence of health professionals and HEWs, women in rural areas choose TBAs first. A previous study conducted in Ethiopia showed that most home births were assisted by TBAs [39]. In the manner now being indicated, TBAs provide services at home and involve in traditional methods that fit into community practice norms, such as burying placenta inside the home. Even some community members believe TBAs are more capable than HEWs [40]. This could be because most women in rural areas trust the TBAs. A lack of trust in the competence of HEWs and limited coordination between them and other healthcare providers contributes to TBAs being the choice of birth attendants in the rural community [41]. Other studies conducted in Ethiopia and Ghana recognized the importance of integrating TBAs into formal health systems, given the lack of maternal health personnel to provide psychosocial support and counseling to women during pregnancy, childbirth, and postpartum in rural and deprived communities [42, 43]. The reason for choosing to give birth at home is that women have perceived or experienced disrespect and abuse by healthcare providers during childbirth [44, 45]. In this regard, strengthening respectful maternity care services throughout the continuum of care and providing training for healthcare providers regarding respectful maternity care can help increase women's preference to use MHSs.

Women's age was a significant and independent factor of intention to use MHSs. In this study, older women (21–30 and > 30-year-old) were 6.04 and 2.57 times more likely intended to use MHSs as compared to their younger women. This finding was in agreement with studies conducted in Kenya [29] and Zambia [36]. This could be because experience in pregnancy and pregnancy-related complications increases as women's age advances. Such 's rich experiences could help them visit health facilities, thereby gaining a comprehensive and favorable awareness of MHSs, which continues to increase their intention to use MHS. Besides, older women could have higher decision-making power with respect to MHSs than younger women [46]. Some evidence support that the use of antenatal care and facility delivery increases with increasing maternal age [47, 48]. In this regard, the policymakers, program managers, and healthcare providers play a role in awareness creation in the communities for policies prioritizing those disadvantaged groups. As a result, they have access to the healthcare services which all mothers deserve.

The current study also revealed that the odds of having an intention to use MHSs among women who had positive subjective norms were 11 times higher than those who had negative subjective norms. This finding is consistent with previous studies conducted in Ethiopia [26, 49]. This suggests that the intention to use MHSs is likely to influence those deemed important to women. Hence, interventions that will address the involvement of people important to women, including husbands/spouses, and other family members such as fathers, mothers, mother in law, father in law, and sisters, would be helpful to enhance the intention of women to use MHSs, thereby increasing the utilization of MHSs. In contrast, studies conducted in Wollaita Soddo, Ethiopia, and Zambia have shown that subjective norms hurt women's intention to use MHSs [36, 38]. The difference might be related to the time gap and study area. Because maternal and child health is one of the top priorities worldwide, a lot of work has been done to raise women's awareness of MHS and improve its utilization.

Our findings indicated that those women who had complications during previous births were 4.6 times more likely to use MHSs for the next pregnancy. Similar findings in Ethiopia reported that complications during a previous pregnancy and childbirth have enabled women to recognize the danger signs of pregnancy and the benefits of skilled birth attendants [26, 50, 51]. This might be as women who had a history of previous negative pregnancy experiences may have better anticipation of pregnancy-related complications than women with no experience. Thus, women experiencing one of the danger signs of pregnancy or adverse pregnancy outcomes may visit a health facility to obtain screening and treatment services. Consequently, they could get information regarding the danger signs of pregnancy and the importance of utilizing MHSs for subsequent pregnancies. Besides, if a woman has dangerous signs of pregnancy and other adverse pregnancy outcomes, she will likely intend to use MHSs for the worry of recurrence and wish for better results. Lastly, the odds of intention to use MHSs among women who had mass media exposure were two times higher than those women who had no mass media exposure. The possible explanation could be that information, education, and communication regarding maternal, neonatal, and child health improvement disseminated through different mass media is one of the government's agendas. This finding is supported by evidence observed from other studies in Ethiopia [39, 52]. Furthermore, the evidence from this study showed that the number of women seeking care during pregnancy and childbirth is still low. So, access to education can significantly improve their maternal health service uptake, which plays a crucial role in reducing maternal morbidity and mortality.

### Limitation of this study

This study has some limitations; first, the source of data for this study was based on the self-reporting of respondents in which recall bias might be introduced. However, this interestingly new finding will offer primary evidence for any health education, counseling provision, community mobilization, and integrating interventions, including behavioral, social, policy, cultural, or other interventions to improve maternal healthcare service utilization within the community context.

## Conclusion

The intention to use MHSs among women who gave birth at home is relatively low in the study setting. Older-aged women (21–30 and > 30-year-old), TBA for the preceding delivery, time to reach health a facility, obstetric danger signs in the recent delivery, positive subjective norms, and mass media exposure were found to be significantly associated with intention to use MHSs in the future. In this regard, community-based interventions like health education on obstetric danger signs and MHSs by emphasizing the benefits of using skilled birth attendants for delivery would be helpful. Besides, strengthening access to transportation, health information communication mass media will enhance the intention to use MHSs. In addition, exploring barriers and facilitators of deep-rooted socio-cultural and behavioral factors in this sub-theme would be helpful.

## Supplementary Information

**Additional file 1.** Knowledge of maternal health services assessment questions.

**Additional file 2.** English version questionnaire.

## Abbreviations

AOR: Adjusted Odds Ratio
ANC: Antenatal Care
HEWs: Health Extension Workers
MMR: Maternal Mortality Ratio
MHS: Maternal Health Service
MWH: Maternity Waiting Home
PNC: Postnatal Care
SPSS: Statistical Package for Social Science
TBA: Traditional Birth Attendants
WHO: World Health Organization
