# Supplementary material for: Intention to use maternal health services and associated factors among women who gave birth at home in rural Sehala Seyemit district: a community-based cross-sectional study
Source: BMC Pregnancy Childbirth. 2022 Mar 16;22:213. doi: 10.1186/s12884-022-04447-y (PMC8928666; doi:10.1186/s12884-022-04447-y)
Supplement: Supplementary file 1 — Additional file 1. Knowledge of maternal health services assessment questions. [file 12884_2022_4447_MOESM1_ESM.docx]

1) Starting early ANC is important

2) Pregnant women may have problems without ANC

3) ANC has to be recommended regardless of complications

4) Regular ANC medications can promote optimal growth of the unborn fetus

5) ANC can prevent complications

6) Do you know when to start ANC

7) At which stage of fetal pregnancy deformities most likely occur?

8) The recommended number of ANC

9) what are malaria prevention methods during pregnancy?

10) What is the prevention of anemia during pregnancy?

11) What is the prevention of parasitic intestinal infection during pregnancy?

12) How can tetanus be prevented during pregnancy?

13) What are the danger signs during pregnancy, intrapartum or postpartum period?

14) What are the components of birth preparedness and complication readiness?

15) What complications will women face without using maternal health services?

16) How many PNC visits will women have?
