# Supplementary material for: Intention to use maternal health services and associated factors among women who gave birth at home in rural Sehala Seyemit district: a community-based cross-sectional study
Source: BMC Pregnancy Childbirth. 2022 Mar 16;22:213. doi: 10.1186/s12884-022-04447-y (PMC8928666; doi:10.1186/s12884-022-04447-y)
Supplement: Supplementary file 2 — Additional file 2. English version questionnaire. [file 12884_2022_4447_MOESM2_ESM.docx]

**English version questionnaire**

**Title**: Intention to use maternal health services and associated factors among women who gave birth at home in rural Sehala Seyemit district: A Community-based cross-sectional study English version of the questionnaire

Principal investigator: Birhan Tsegaw Taye

Co-authors: Azmeraw Ambachew Kebede

Kindu Yinges Wondie

Part I: Socio-demographic characteristics

| N.O | Questionnaire | Alternative choice for response | Skip code |
| --- | --- | --- | --- |
| 101 | How old are you? | (In years) |  |
| 102 | What is your educational level? | 1. Can’t read and write  2. Can read and write  3. Primary  4. Secondary  5. Diploma and above |  |
| 103 | What is your marital status? | 1. Single  2. Married  3. Divorced  4. Widowed  5. Separated |  |
| 104 | What is your occupation? | 1.House wife  2. Merchant  3. Government employee  4. self employed  5. Student  6. Others specify…………………………. |  |
| 105 | What is your religion? | 1. Orthodox Christian  2. Muslim  3. Protestant  4. Catholic  5. Other specify…………………………. |  |
| 107 | How many persons are living at home? |  |  |
| 108 | What is your husband educational level? | 1. Can’t read and write  2. Can read and write  3. Primary  4. Secondary  5. Diploma and above |  |
| 109 | What is your husband occupation? | 1. Daily labor 2. Farmer 3. Merchant 4. Government employee 5. Self employed 6. Student 7. Others specify…………………………. |  |
| 110 | Have you ever read newspapers? | 1. Yes 2. No | If yes got to Q 111 |
| 111 | If yes, how often did you read? | 1. Every day 2. One times per week 3. Two times per week 4. Three and more |  |
| 112 | Have you ever heard radio? | 1. Yes 2. No | If yes got to Q 113 |
| 113 | If yes, how often did you heard? | 1. Every day 2. One times per week 3. Two times per week 4. Three and more |  |
| 114 | How long does it take from your home to the nearby health facility on foot? | 1. 1. < 1 hour   2. ≥ 1 hour |  |

Part II: Reproductive and maternity health service related characteristics

| NO | Questioners | Alternative choices for response | Skip code |
| --- | --- | --- | --- |
| 201 | How many times did you give birth after 7 months? |  |  |
| 202 | Did you have ANC follow-up in your most recent pregnancy? | 1. Yes  2. No | If yes got to 203 |
| 203 | How many ANC visits did you had on your most recent pregnancy? | 1. Once  2. Twice  3. Three times  4. Four and above |  |
| 204 | Who assisted your most recent delivery? | 1. 1. HEWs 2. 2. TBA 3. 3. Family |  |
| 205 | Did you have PNC visit in your most recent child? | 1. 1. Yes 2. 2. No |  |
| 206 | How many PNC visits did you have? | 1. 1. One 2. 2. Two 3. 3. Three or more |  |
| 208 | Did you have a history of neonatal death? | 1. 1. Yes   2. No |  |
| 209 | Do you have a history of abortion? | - - - 1. Yes       2. No |  |
| 210 | Do you have history of obstetric danger signs in your most recent pregnancy? | 1. 1. Yes 2. 2. No |  |
| 211 | Husband/Partner involvement related questions on maternal and child health related services | | |
| I | Did your husband go with you for ANC follow up at least once in your most recent pregnancy? | 1. Yes  2. No |  |
| II | Did your husband provide transport/gave money for transport during your recent pregnancy or delivery? | 1.Yes  2. No |  |
| III | Did your husband accompany to the hospital during labor for your recent delivery? | 1. Yes  2. No |  |
| IV | Did your husband discuss with health care providers during your recent pregnancy or delivery? | 1. Yes  2. No |  |
| V | Did your husband look after the child at home/stay with babies while you are outside home? | 1. Yes  2. No |  |
| VI | Did your husband bath newborn/infant while you are busy? | 1. Yes  2. No |  |
| VII | Did your husband buy clothes/other things for infants/neonates? | 1. Yes  2. No |  |
| VIII | Did your husband go with you for immunization services? | 1. Yes  2. No |  |
| IX | Did your husband assisted you while you breastfeed the newborn? | 1. Yes  2. No |  |
| 212 | Was your most recent pregnancy planned? | 1. 1. Yes 2. 2. No |  |
| 213 | Why you didn’t use antenatal care and institutional delivery for your most recent pregnancy? |  |  |

Part III: Mothers knowledge related questions on maternal health service

| No | Questionnaire | Alternative choice for response | Skip code |
| --- | --- | --- | --- |
| 301 | Have you ever heard about ANC and PNC? | 1. 1.Yes 2. 2. No |  |
| 302 | Do you know when to start the first ANC visit? | 1. 1. Before 3 months 2. 2. 3 to 4 months 3. 3. 4 to 5 months 4. 4. Other specify |  |
| 303 | Do you think ANC and PNC can prevent complications? | 1. 1. Yes 2. 2. No |  |
| 304 | Pregnant women may have problems without ANC? | 1. 1. Yes 2. 2. No |  |
| 305 | Regular ANC medications can promote optimal growth of unborn child? | 1.Yes  2. No |  |
| 306 | Health facility delivery is safer and better than home delivery? | 1.Yes   1. 2. No |  |
| 307 | Do you think starting ANC and PNC early will be important? | 1.Yes   1. 2. No |  |
| 308 | Do you know the recommended frequency of ANC visits? |  |  |
| 309 | Do you think ANC and PNC has to be recommended regardless of complications? | 1.Yes  2. No |  |
| 310 | At which stage of pregnancy fetal deformity most likely occur? | 1. Below 3^rd^ moth 2. Between the 3^rd^ and the 7^th^ month 3. After the 7^th^ month 4. Don’t know |  |
| 311 | Perception of first fetal movement | 1. At the 3^rd^ month 2. Between the 4^th^ and 5^th^ month 3. Don’t know |  |
| 312 | When we say a women have optimum ANC? | 1. 4 and above 2. Less than 4 |  |
| 313 | Do you think maternal waiting homes are important for a pregnant women? | 1.Yes  2. No |  |
| 314 | Do you know the obstetric danger signs during pregnancy and postpartum period? | 1.Yes  2. No |  |
| 315 | If yes for Q319, list all the obstetric danger signs during pregnancy and postpartum period | 1. Vaginal bleeding 2. Blurring of vision 3. Epigastric pain 4. High grade fever 5. Convulsion 6. Loss of consciousness 7. Decreased/absent fetal movement 8. Other specify |  |
| 316 | Do you know smoking cigarate and drinking alcohol is unsafe for the fetus? | 1. Yes 2. No |  |
| 317 | Do you how to prevent malaria during pregnancy | 1. Use of insecticide treated nets 2. Eliminating stagnant water 3. Use of antimalarial prophylaxis 4. Other specify |  |
| 318 | Do you know how to prevent anemia during pregnancy? | 1. Use of iron as recommended 2. Avoid milk, coffee and tea with meals 3. Eating green leafy vegetables 4. Don’t know |  |
| 319 | Do you know how to prevent helminthic infection during pregnancy? | 1. Use of mebendazole 2. Avoid eating raw meat 3. Avoid bare foot 4. Don’t know |  |
| 320 | Do you how to prevent tetanus during pregnancy? | 1. Use of tetanus toxoid vaccine 2. Don’t know |  |
| 321 | What complications a woman will face without ANC, facility delivery and PNC? |  |  |
| 322 | Do know the components of birth preparedness and complication readiness | 1. Identify qualified birth attendants 2. Identify health facility for delivery 3. Arranged transport for emergency 4. Saving money 5. Prepare blood donor 6. Prepare materials for delivery 7. Identify danger signs and ready for complication 8. I don’t know |  |
| Part Iv: Attitude related questions | | | |
| 401 | All pregnant women should have ANC and PNC follow up | Strongly agree  Agree  Neutral  Disagree  Strongly disagree |  |
| 402 | Pregnant and lactating women should use a variety of foods rich in protein and vitamins | Strongly agree  Agree  Neutral  Disagree  Strongly disagree |  |
| 403 | The healthcare provided by health providers is important | Strongly agree  Agree  Neutral  Disagree  Strongly disagree |  |
| 404 | Timely ANC and PNC follow-up will be safer for both mother and baby during labor and delivery | Strongly agree  Agree  Neutral  Disagree  Strongly disagree |  |
| 405 | Taking medication during pregnancy without a doctor's prescription can cause problems for the fetus | Strongly agree  Agree  Neutral  Disagree  Strongly disagree |  |
| 406 | Husbands should be present during ANC, delivery and PNC | Strongly agree  Agree  Neutral  Disagree  Strongly disagree |  |
| 407 | It is important to be prepared during pregnancy | Strongly agree  Agree  Neutral  Disagree  Strongly disagree |  |
| 408 | Maternal waiting homes are very important for women far from health facilities | Strongly agree  Agree  Neutral  Disagree  Strongly disagree |  |
| 409 | Heavy weight lifting and strenuous exercise during pregnancy is dangerous and may be un safe for the fetus | Strongly agree  Agree  Neutral  Disagree  Strongly disagree |  |
| 410 | Advice regarding proper health during pregnancy and child birth can be gotten outside the hospital | Strongly agree  Agree  Neutral  Disagree  Strongly disagree |  |
| 411 | Follow up during pregnancy may decrease intrapartum and postpartum complications | Strongly agree  Agree  Neutral  Disagree  Strongly disagree |  |
| 412 | Health facility delivery is safer and better than home delivery | Strongly agree  Agree  Neutral  Disagree  Strongly disagree |  |
| 413 | Women may have problems without ANC, health facility delivery and PNC | Strongly agree  Agree  Neutral  Disagree  Strongly disagree |  |

Part V: Decision making related questions

| No | Questionnaire | Alternative choice for response | Skip code |
| --- | --- | --- | --- |
| 501 | Who decide about health care for yourself? | 1. Me alone 2. My husband alone 3. Both of us 4. Other specify |  |
| 502 | Who decides on large household purchase or sell? | 1. Me alone 2. My husband alone 3. Both of us 4. Other specify |  |
| 503 | Who decides on intrahousehold resource allocation/ daily household purchases? | 1. Me alone 2. My husband alone 3. Both of us 4. Other specify |  |
| 504 | Who decides on where and when to seek medical care for sick newborns/children? | 1. Me alone 2. My husband alone 3. Both of us 4. Other specify |  |
| 505 | Who decides on visits of family, friends or relatives? | 1. Me alone 2. My husband alone 3. Both of us 4. Other specify |  |
| 506 | Who decides when to have an additional child? | 1. Me alone 2. My husband alone 3. Both of us 4. Other specify |  |
| 507 | Who usually decides how your partner’s/husband earnings will be used? | 1. Me alone 2. My husband alone 3. Both of us 4. Other specify |  |
| 508 | Who decides to go for ANC visit, PNC visit, where to deliver and infant immunization? | 1. Me alone  2. My husband alone  3. Both of us  4. Other specify |  |
| 509 | Who usually decides what foods to be cooked each day? | 1.Me alone  2.My husband alone  3.Both of us  4. Other specify |  |

Part VI: Women’s intention to use maternal health services

| S.no | Question | Answer |
| --- | --- | --- |
| 601 | Intention to use antenatal care | 1. Strongly agree 2. Agree 3. Neutral 4. Disagree 5. Strongly disagree |
| 602 | Intention to use maternal waiting homes | 1. Strongly agree 2. Agree 3. Neutral 4. Disagree 5. Strongly disagree |
| 603 | Intention to pay for ANC/ delivery if it is with a cost | 1. Strongly agree 2. Agree 3. Neutral 4. Disagree 5. Strongly disagree |
| 604 | Intention to deliver in a health facility | 1. Strongly agree 2. Agree 3. Neutral 4. Disagree 5. Strongly disagree |
| 605 | Intention to use postnatal care | 1. Strongly agree 2. Agree 3. Neutral 4. Disagree 5. Strongly disagree |

Part VII: Subjective norms and perceived behavioral control related questions

| **Subjective norms** | | |
| --- | --- | --- |
| 701 | People who are important to me think that I should use antenatal care during pregnancy | 1. Strongly agree 2. Agree 3. Neutral 4. Disagree 5. Strongly disagree |
| 702 | Important people to me think that I should use maternal waiting homes in the last 2-4 weeks of my pregnancy | 1. Strongly agree 2. Agree 3. Neutral 4. Disagree 5. Strongly disagree |
| 703 | People who are important to me think that I should deliver in a health facility | 1. Strongly agree 2. Agree 3. Neutral 4. Disagree 5. Strongly disagree |
| 704 | Important people to me think that I should get a skilled birth attendant during delivery | 1. Strongly agree 2. Agree 3. Neutral 4. Disagree 5. Strongly disagree |
| 705 | Important people to me think that I should follow postnatal care and immunization services | 1. Strongly agree 2. Agree 3. Neutral 4. Disagree 5. Strongly disagree |
| **Perceived behavioral control** | | |
|  | For me attending antenatal care is simple and I can do it | 1. Strongly agree 2. Agree 3. Neutral 4. Disagree 5. Strongly disagree |
| 707 | For me using maternal waiting homes in the last 2-4 weeks of my pregnancy is simple and I can do it | 1. Strongly agree 2. Agree 3. Neutral 4. Disagree 5. Strongly disagree |
| 708 | For me health facility delivery is very simple and I can do it | 1. Strongly agree 2. Agree 3. Neutral 4. Disagree 5. Strongly disagree |
| 709 | Getting a skilled birth attendant is easy for me and I can do it | 1. Strongly agree 2. Agree 3. Neutral 4. Disagree 5. Strongly disagree |
| 710 | Using postnatal and immunization services is easy to me and I can do it | 1. Strongly agree 2. Agree 3. Neutral 4. Disagree 5. Strongly disagree |
